# Multilokuläres Pyoderma gangraenosum: Assoziation mit der Erstmanifestation einer primär biliären Cholangitis

**DOI:** 10.1007/s00105-023-05161-2

**Published:** 2023-06-16

**Authors:** Julian Kögel, Mark Berneburg, Sigrid Karrer, Konstantin Drexler, Dennis Niebel

**Affiliations:** Klinik und Poliklinik für Dermatologie und Allergologie, Universitätsklinikum Regensburg, Franz-Josef-Strauß-Allee 11, 93053 Regensburg, Deutschland

**Keywords:** Sweet-Syndrom, Neutrophile Dermatose, Primär biliäre Cholangitis, PAPA-Syndrom, Ulzeration, Sweet’s syndrome, Neutrophilic dermatosis, Primary biliary cholangitis, PAPA syndrome, Ulceration

## Abstract

Vorstellung einer 16-jährigen Patientin mit vorbekannter Acne vulgaris in reduziertem Allgemeinzustand mit akut aufgetretenen, schmerzhaften Ulzerationen. Die Infektparameter zeigten sich stark erhöht, es bestand kein Fieber. Wir stellten die Diagnose eines multilokulären Pyoderma gangraenosum. Nebenbefundlich konnte eine primär biliäre Cholangitis diagnostiziert werden. Wir führten eine systemische Kortikosteroidtherapie durch sowie eine Therapie mit Ursodesoxycholsäure. Hierunter kam es zu einer raschen Besserung. Ein PAPA-Syndrom konnte humangenetisch ausgeschlossen werden.

## Abstract (en)

A 16-year-old female patient with previously diagnosed acne vulgaris was transferred to our clinic in reduced general condition with rapidly progressive and extremely painful ulcerations. In the laboratory exam, inflammatory parameters were highly elevated, but she was normothermic. Based on the findings, we diagnosed multilocular pyoderma gangrenosum. Further investigations established the diagnosis of primary biliary cholangitis as the underlying condition. Treatment with systemic corticosteroids was initiated and we started therapy with ursodeoxycholic acid. This led to improvement within a few days. PAPA-syndrome (pyogenic arthritis, pyoderma gangrenosum and acne vulgaris) could be ruled out by genetic analysis.

## Anamnese

Eine 16-jährige Patientin stellte sich mit zunehmenden brennenden Hautveränderungen im Bereich des Rückens, des Dekolletés und Gesichts zunächst in der örtlichen Kinderklinik vor. Seit mehreren Jahren war eine Acne vulgaris vorbekannt, die unter anderem mittels Isotretinoin systemisch behandelt wurde. Aufgrund des nun vorliegenden Hautbefundes erfolgte zunächst die Therapie mittels Cefuroxim intravenös. Bei progredientem Hautbefund und Anstieg der Entzündungsparameter erfolgte die Zuverlegung an unsere Klinik.

## Befund

**Figure 1:**
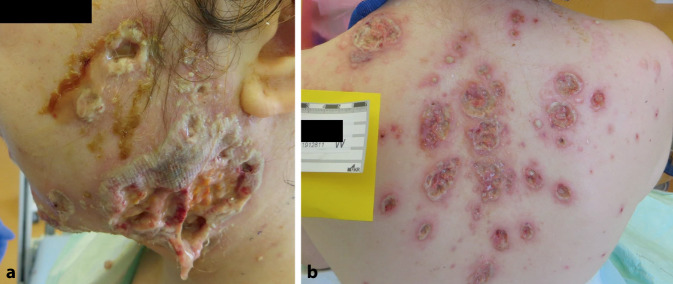

Klinisch bestanden bei Aufnahme an den Wangen beidseits, am Dekolleté und am Rücken stellenweise bis auf das subkutane Fettgewebe reichende, rundlich ovale, eher scharf begrenzte Ulzerationen mit erythematösem, teils unterminiertem Randsaum. Die Ulzerationen waren eitrig, schmierig belegt. Im Gesichtsbereich zeigten sich auch gelbliche Krusten. Am Rücken kamen einzelne Pusteln zur Darstellung (Abb. 1). Die Patientin wies einen deutlich reduzierten Allgemeinzustand auf, es bestanden ausgeprägte Schmerzen im Bereich der betroffenen Areale. Es bestanden keine Gelenkschmerzen und kein Fieber.

Die Infektparameter stellten sich stark erhöht dar (Tab. 1). Weitere Laborparameter können Tab. 1 entnommen werden.

**Figure 2:**
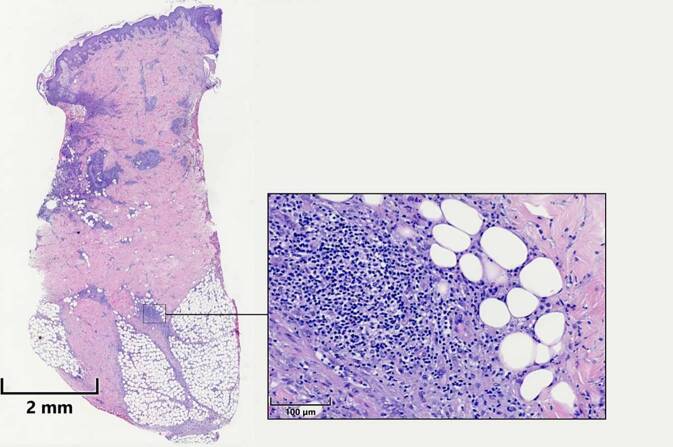

- Blutausstrich: keine Blasten oder atypische Zellen
- Abdomensonographie: zentrale Gallenwege betont darstellbar mit echoreichem verdicktem Randsaum
- Gastroskopie: leichte, nicht erosive Typ-C-Gastritis und Duodenitis
- Koloskopie: leicht floride Proktitis
- Mikrobiologische Untersuchungen (Abstriche und Stanzbiopsat): keine bakterielle Besiedelung
- Histologie Stanzbiopsien (Abb. 2):
  - Randbereich einer Ulzeration: akanthotisch verbreiterte, von neutrophilen Granulozyten durchsetzte Epidermis. Bis ins subkutane Fettgewebe reichende Infiltrate aus fast ausschließlich neutrophilen Granulozyten
  - Frische Pustel: Infiltrate von neutrophilen Granulozyten bis ins mittlere Korium, dazwischen Reste kleiner Epithelinseln und teils Erythrozytenextravasation. Im Randbereich zeigt sich eine Akanthose mit subepidermalem Ödem
- Humangenetische Untersuchung: Ausschluss eines PAPA-Syndroms und adrenogenitalen Syndroms (Genpanel s. Tab. 2)
- Paracelsus-Score: 18 Punkte (Tab. 3)

**Table 1:** 

Laborparameter	Befund	Referenzbereich
C‑reaktives Protein	*420* *mg/l*	< 5 mg/l
Procalcitonin	*2,52* *ng/ml*	< 0,06 ng/ml
Leukozyten	*73,80/nl*	4,23–9,1/nl
Neutrophile Granulozyten	*64,58/nl* *87,6* *%*	1,78–5,38/nl 37–75 %
Lymphozyten	*0,79/nl* *1,1* *%*	1,0–3,2/nl 14–48 %
ANA-Titer	*1:1280*	< 1:80
Ds-DNA-AK	< 9,8 IU/ml	< 27 IU/ml
DFS-70-AK	*450,8 CU*	< 20 CU
cANCA-AK	*15,9* *IU/ml*	< 5 IU/ml
pANCA-AK	< 1,0 IU/ml	< 6 IU/ml
Scl 70-AK	Negativ	–
SS-A-AK	Negativ	–
SS-B-AK	Negativ	–
Sm-AK	Negativ	–
RNP-AK	Negativ	–
PML-AK	*Stark positiv*	–
Cardiolipin-AK (IgG und IgM)	Unauffällig	–
Beta2 – Glykoprotein-AK (IgG und IgM)	Unauffällig	–
C3c	81,9 mg/dl	60–180 mg/dl
C4	21,4 mg/dl	20–40 mg/dl
Calprotectin im Stuhl	*1375,7* *mg/kg*	< 50 mg/kg

*AK* Antikörper

**Table 2:** 

CYP11B1	CYP11B2	CYP17A1	CYP21A2	C7
HSD3B2	IL1RN	MEFV	NCSTN	NFKB1
NLRP3	NOD2	PLCG2	PSMB8	PSTPIP1

**Table 3:** 

	Punktwert
*Hauptkriterium (3 Punkte)*	
Progredienter Krankheitsverlauf	3
Ausschluss relevanter Differenzialdiagnosen	3
Rötlich livider Wundrand	3
*Nebenkriterium (2 Punkte)*	
Ansprechen auf Immunsuppressiva	2
Charakteristisch bizarre Form der Ulzeration	2
Extremer Schmerz VAS > 4	2
Lokales Pathergiephänomen	–
*Zusatzkriterium (1 Punkt)*	
Suppurative Inflammation in der Histologie	1
Unterminierter Randsaum	1
Systemerkrankung assoziiert	1
**Gesamt:**	18

*VAS* Visuelle Analogskala

## Diagnose

Multilokuläres Pyoderma gangraenosum (PG) in Assoziation mit der Erstmanifestation einer primär biliären Cholangitis (PBC).

## Therapie und Verlauf

Wir begannen eine systemische Kortikosteroidtherapie mit 60 mg Prednisolon i.v. (entsprechend 1 mg/kgKG). Analgetisch erhielt die Patientin Nalbuphin und Metamizol i.v. Die Wundversorgung erfolgte mittels Polihexanid-Wundgel, Diflucortolon-21-pentanoat topisch im Randbereich der Ulzerationen sowie Fettgaze und absorbierenden Schaumstoffverbänden. Bei serologischem und sonographischem Verdacht auf eine PBC wurde eine Therapie mit Ursodesoxycholsäure eingeleitet.

**Figure 3:**
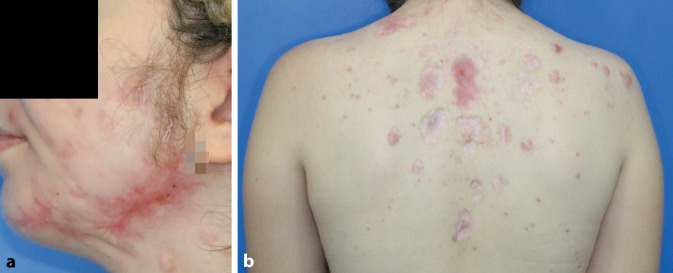

Unter der stationären Therapie kam es bereits innerhalb weniger Tage zur Besserung des Hautbefundes und zu einer deutlichen Reduktion der Schmerzen. Im Labor zeigte sich ein deutlicher Rückgang der Neutrophilie. Im ambulanten Bereich konnte die Prednisolon-Therapie ausgeschlichen und schließlich komplett beendet werden. Ein Rezidiv der Erkrankung blieb bis mittlerweile mehr als ein Jahr nach Diagnosestellung aus. Die Hautveränderungen heilten narbig ab (Abb. 3). Aktuell erfolgt die Anwendung eines silikonhaltigen Narbengels.

## Diskussion

Systemerkrankungen wie chronisch entzündliche Darmerkrankungen, rheumatoide Arthritis und hämatologische Erkrankungen können mit einem PG assoziiert sein [7]. Ein gemeinsames Auftreten mit einer PBC, wie es in der von uns dargestellten Kasuistik der Fall ist, wurde bisher selten beschrieben [5].

Beim PG können erhöhte Entzündungswerte imponieren, Fieber tritt meist nicht auf [7, 8]. Aufgrund der bei Aufnahme bestehenden Leukozytose mit absoluter Neutrophilie und dem raschen Ansprechen auf die Glukokortikoidtherapie diskutierten wir differenzialdiagnostisch ein bullöses Sweet-Syndrom [9]. Die in unserem Fall vorliegenden tiefen Ulzerationen wurden beim bullösen Sweet-Syndrom bisher jedoch nicht beschrieben, auch das Fehlen von Fieber ist für das Sweet-Syndrom untypisch [4, 10]. Der klinische Befund in Zusammenhang mit erhöhten Entzündungsparametern lässt differenzialdiagnostisch an eine Pyodermie denken. Das fehlende Ansprechen auf die initiale Antibiotikatherapie, der fehlende Bakteriennachweis in den entnommenen Abstrichen und Gewebebiopsaten sowie die ausgeprägte Schmerzhaftigkeit der Läsionen in Kombination mit einer allzeit afebrilen Patientin führten uns jedoch zur Ausschlussdiagnose eines multilokulären PG. Die aktuelle AWMF-Leitlinie empfiehlt zur Diagnostik eines PG den Paracelsus-Score [1]. Der in unserem Fall vorliegende Punktwert von 18 macht die Diagnose eines PG sehr wahrscheinlich. Sowohl Sweet-Syndrom als auch PG werden dem engeren Formenkreis der neutrophilen Dermatosen zugeordnet, wobei es jeweils zu kutanen Infiltraten von neutrophilen Granulozyten kommt. Sowohl beim PG als auch beim Sweet-Syndrom konnten erhöhte Spiegel von Interleukin-1β nachgewiesen werden [6]. In der Literatur werden Übergangsformen zwischen Sweet-Syndrom und PG beschrieben [4, 10].

Autoinflammationssyndrome wie PAPA (pyogene Arthritis, PG, Acne vulgaris), PASH (PG, Acne vulgaris, Hidradenitis suppurativa) und PA-PASH (pyogene Arthritis, Acne vulgaris, PG, Hidradenitis suppurativa) gehen definitionsgemäß mit dem Auftreten eines PG einher. Pathophysiologische Grundlage ist eine Überaktivierung des angeborenen Immunsystems, die zu einer Überproduktion von vorwiegend Interleukin-1β und TNF‑α führt. Beim PAPA-Syndrom ist eine autosomal-dominant vererbte Mutation im *PSTPIP1*-Gen beschrieben [2]. Eine humangenetische Untersuchung bei klinischem Verdacht ist daher indiziert.

Aufgrund überlappender Pathomechanismen werden neutrophile Dermatosen wie PG und Sweet-Syndrom als kutane Manifestationen einer Autoinflammation betrachtet [6]. Die Therapie erfolgt entsprechend immunsuppressiv.

## Fazit für die Praxis

- Das Auftreten eines PG kann in seltenen Fällen mit einer PBC assoziiert sein.
- Die Abgrenzung eines PG zu Differenzialdiagnosen kann schwierig sein, eine rasche immunsuppressive Therapie ist jedoch entscheidend.
- Autoinflammationssyndrome (PAPA, PASH, PA-PASH) sollten bei Diagnose eines PG im Kindes- und Jugendalter berücksichtigt werden, v. a. bei Vorliegen von Komorbiditäten in Form von Acne vulgaris, Gelenkschmerzen oder Hidradenitis suppurativa.

## Abkürzungen

AK: Antikörper
PBC: Primär biliäre Cholangitis
PG: Pyoderma gangraenosum
VAS: Visuelle Analogskala
